# TP53 mutations determined by targeted NGS in breast cancer: a case-control study

**DOI:** 10.18632/oncotarget.28071

**Published:** 2021-10-12

**Authors:** Angeliki Andrikopoulou, Evangelos Terpos, Spyridoula Chatzinikolaou, Kleoniki Apostolidou, Ioannis Ntanasis-Stathopoulos, Maria Gavriatopoulou, Meletios-Athanasios Dimopoulos, Flora Zagouri

**Affiliations:** ^1^Department of Clinical Therapeutics, Alexandra Hospital Medical School, Athens 11528, Greece

**Keywords:** TP53 mutations, next-generation sequencing, biomarker, prognosis, breast cancer

## Abstract

Background: Tumor protein 53 (TP53) gene mutations are identified in up to 37% of breast tumors especially in HER-2 positive and basal-like subtype. Previous studies have indicated TP53 mutations as a prognostic biomarker in breast cancer. However, most of these studies performed immunohistochemistry (IHC) for the detection of TP53 mutations.

Aim: The purpose of our study is to evaluate the role of TP53 somatic mutations detected via next-generation sequencing (NGS) as a potential prognostic marker in patients with breast cancer.

Materials and Methods: 82 female patients with Stage I–III breast cancer underwent NGS in paraffin blocks and blood samples during the period 25/09/2019 through 25/05/2021. 23 cases of somatic TP53 mutations and 23 cases of healthy controls were matched on age at diagnosis, menopausal status, histological subtype, histological grade, ki67 expression and disease stage.

Results: Mean age at diagnosis was 52.35 (SD; 11.47) years. The somatic TP53 mutation *NM_000546.5:c.824G>A p.(Cys275Tyr)* was most frequently detected. Co-existence of PIK3CA mutation was a common finding in somatic TP53-mutant tumors (4/23; 17.4%). Disease-free survival was shorter in TP53-mutated cases (16.3 months vs. 62.9 months). TP53 pathogenic somatic mutations were associated with a 8-fold risk of recurrence in the univariate Cox regression analysis (OR = 8.530, 95% CI: 1.81–40.117; *p* = 0.007).

Conclusions: Our case-control study suggests that TP53 somatic mutations detected by next-generation sequencing (NGS) are associated with an adverse prognosis in breast cancer.

## INTRODUCTION

TP53 gene is the most frequently mutated gene (>50%) in human cancer, indicating its crucial role as a tumor suppressor [1]. TP53 gene encodes p53 protein which is considered as “the guardian of the genome” by binding to specific DNA sequences and maintaining genomic stability [2]. P53 protein is involved in cell response to stress signals, activates DNA repair proteins and regulates the production of stem cells [3]. The fundamental role of TP53 is evident in Li-Fraumeni syndrome which is characterized by germline mutations of TP53 and predisposition to aggressive tumors like early-onset breast cancer (25%), soft-tissue or bone sarcomas (35%) and brain tumors. In breast cancer, germline TP53 mutations harbor 5–8% of cases of early-onset (under 30 years old) disease, while up to 85% of women who carry germline TP53 mutations eventually develop breast cancer [4]. On the other hand, somatic TP53 mutations are identified in 37% of all breast cancers and is more frequently mutated in HER-2 positive (72%) and basal-like subtype (80%) [5]. DNA sequencing is considered as the gold-standard for the detection of TP53 mutations that usually harbor the exons 5–8 of the gene [6]. More recently, next generation sequencing has allowed the detection of TP53 mutations outside this restricted region.

There are numerous studies assessing the clinical significance of TP53 mutations in breast cancer. However, the results of these studies are often contradicting. TP53 mutation was associated with worse prognosis in breast cancer patients regardless of the tumor subtype and the type of treatment administered. Initially, p53 IHC expression was associated with an adverse prognosis in small retrospective studies [7–9]. More recent studies evaluated TP53 gene mutations in breast cancer via DNA sequencing [10–13]. P53 mutation was associated with negative estrogen and progesterone receptor (ER/PR) status and increased mortality rate in 859 breast cancer women [10]. TP53 mutations within exons 5 to 8 detected by gene sequencing were related to increased risk of breast cancer-specific death regardless of tumor size, nodal status and hormone receptor expression [11]. Moreover, P53 mutation status has been associated with response to breast cancer treatment. A METABRIC analysis of breast cancer patients that received only endocrine treatment linked *TP53* mutations to worse survival [6], while a meta-analysis of 3,476 cases of patients receiving neoadjuvant treatment concluded that TP53 mutation status is a predictor of response to neoadjuvant chemotherapy [14].

The aim of this study is to evaluate the role of somatic TP53 mutations as a potential prognostic marker in Stage I-III breast cancer patients treated in a single center via a case–control study. The identification of TP53 mutations was performed via next-generation sequencing (NGS) in paraffin blocks of the patients enrolled.

## RESULTS

### Clinicopathological characteristics

Patient characteristics (age, date at diagnosis, menopausal status), histopathological parameters (histological type, grade, ER/PR expression, HER2 expression, ki67, stage) and treatment administered (surgery, chemotherapy, anti-HER2 treatment, hormonotherapy) in cases and controls are summarized in Tables 1 and 2.

**Table 1:** Clinicopathological characteristics of cases and controls

Variable Continuous variables	Cases Mean (SD)	Controls Mean (SD)	*p*-value
**Age at diagnosis**	52.35 (11.47)	49.26 (11.27)	*p* = 0.362
**Menopausal status**			*p* = 0.492
* Premenopausal*	8 (34.8%)	12 (52.2%)	
* Perimenopausal*	4 (17.4%)	3 (13.0%)	
* Postmenopausal*	11 (47.8%)	8 (34.8%)	
**Surgery**			*p* = 0. 295
* Yes*	20 (87.0%)	22 (95.7%)	
* No*	3 (13.0%)	1 (4.3%)	
**Stage at diagnosis**			*p* = 0.802
* I*	5 (21.7%)	4 (17.4%)	
* II*	6 (26.1%)	8 (34.8%)	
* III*	12 (52.2%)	11 (14.8%)	
**Breast tumor location**			*p* = 0.546
* Left*	13 (56.5%)	15 (65.2%)	
* Right*	10 (43.5%)	8 (34.8%)	
**Histology**			*p* = 0.368
* IDC*	22 (95.7%)	22 (95.7%)	
* ILC*	1 (4.3%)	0 (0%)	
* Other*	0 (0.0%)	1 (4.3%)	
**Hormone status**			*p* = 0.760
* Positive*	14 (60.9%)	15 (65.2%)	
* Negative*	9 (39.1%)	8 (34.8%)	
**ER status**			*p* = 0.546
* Positive*	10 (43.5%)	8 (34.8%)	
* Negative*	13 (56.5%)	15 (65.2%)	
**PR status**			*p* = 0.552
* Positive*	12 (52.2%)	14 (60.9%)	
* Negative*	11 (47.8%)	9 (39.1%)	
**HER2 status**			*p* = 0.760
* Positive*	9 (39.1%)	8 (34.8%)	
* Negative*	14 (60.9%)	15 (65.2%)	
**Grade**			
* G1*	1 (4.3%)	0 (0.0%)	*p* = 0.506
* G2*	5 (21.7%)	7 (30.4%)	
* G3*	17 (73.9%)	16 (69.6%)	
***Ki67***			*p* = 0.681
* *<20%	3 (13.0%)	4 (17.4%)	
* *≥20%	20 (87.0%)	19 (82.6%)	

**Table 2:** Type of treatment administered in cases and controls

**Adjuvant chemotherapy**			*p* = 0.636
*No*	3 (13.0%)	2 (8.7%)	
*Yes*	20 (87.0%)	21 (91.3%)	
**Adjuvant radiation**			*p* = 0.743
*No*	6 (26.1%)	7 (30.4%)	
*Yes*	17 (73.9%)	16 (69.6%)	
**Anti-Her2 treatment**			*p* = 0.760
*No*	14 (60.9%)	15 (65.2%)	
*Yes*	9 (39.1%)	8 (34.8%)	
**Disease progression**			
*No*	14 (60.9%)	21 (91.3%)	
*Yes*	9 (39.1%)	2 (8.7%)	

Mean age at diagnosis was 52.35 (SD; 11.47) years in cases and 49.26 years (SD; 11.27) years in controls (*p* = 0.362). Overall, there were not statistically significant differences between cases and controls in terms of age at diagnosis (*p* = 0.362), menopausal status (*p* = 0.492), stage at diagnosis (*p* = 0.802), histology (*p* = 0.368), hormone status (*p* = 0.760), HER2 expression (*p* = 0.760), grade (G1 vs. G2/3; *p* = 0.506) and Ki67 status (*p* = 0.681). Patients with stage I–III disease (23; 100%) underwent surgical excision (20; 87%) and adjuvant radiation (17; 73.9%). Invasive ductal carcinoma (IDC) was diagnosed in most of the cases (22; 95.7%). The majority of cases were hormone receptor-positive (14; 60.9%), HER2-negative (14; 60.9%) and characterized by low differentiation (17; 73.9%) and high Ki67 expression (20; 87%).

### Genetic polymorphisms of TP53 somatic mutations

Genetic polymorphisms of TP53 pathogenic somatic mutations identified are summarized in Table 3. Of note, the most frequent pathogenic somatic TP53 mutations reported in our patients were *c.824G>A p.Cys275Tyr* (*n* = 3) and *c.743G>A p.Arg248Gln* (*n* = 2) while the other polymorphisms were detected only once.

**Table 3:** Genetic polymorphisms of pathogenic somatic TP53 mutations

TP53 genetic polymorphism	NCBI genomes browser	Type	Clinical significance	Frequency
c.614A>G p.Tyr205Cys	Rs1057520007	Somatic	Pathogenic	1
c.559+1G>A	Rs1131691042	Somatic	Pathogenic	1
c.824G>A p.Cys275Tyr	Rs863224451	Somatic	Pathogenic	3
c.488A>G p.Tyr163Cys	Rs148924904	Somatic	Pathogenic	1
c.818G>T p.Arg273Leu	Rs28934576	Somatic	Pathogenic	1
c.714_715insT p.Asn239Ter	Rs1567549651	Somatic	Pathogenic	1
c.536A>G p.His179Arg	Rs1057519991	Somatic	Pathogenic	1
c.85_86del p.Asn29GlnfsTer13	Rs1555526931	Somatic	Pathogenic	1
c.586C>T p.Arg196*	Rs397516435	Somatic	Pathogenic	1
c.797G>A p.Gly266Glu	Rs193920774	Somatic	Pathogenic	1
c.853G>A p.Glu285Lys	Rs112431538	Somatic	Pathogenic	1
c.990del p.Gln331Argfs*14	Rs11575996	Somatic	Pathogenic	1
c.742C>T p.Arg248Trp	Rs121912651	Somatic	Pathogenic	1
c.722C>T p.Ser241Phe	Rs28934573	Somatic	Pathogenic	1
c.743G>A p.Arg248Gln	Rs11540652	Somatic	Pathogenic	2
c.638G>T p.Arg213Leu	Rs587778720	Somatic	Pathogenic	1
c.455C>T p.Pro152Leu	Rs587782705	Somatic	Pathogenic	1
c.817C>T p.Arg273Cys	Rs121913343	Somatic	Pathogenic	1
c.681_682insT p.Asp228Ter	Rs1567550002	Somatic	Pathogenic	1
c.626_627del p.Arg209LysfsTer6	Rs1057517840	Somatic	Pathogenic	1

We examined the presence of other pathogenic mutations along with TP53 mutations. The genomic profile of our cases is summarized in Table 4. Of note, PIK3CA was the most frequent pathogenic mutation detected in somatic TP53-mutant tumors (4/23; 17.4%). Other pathogenic mutations identified included AKT1, PTEN and NRAS mutations. Of note, a number of mutations of unknown significance were frequently reported. The most common mutations of unknown significance (VUS) identified were: ROS1 (10/23), KMT2C (6/23), NF1 (4/23), RET (2/23), NOTCH1 (2/23).

**Table 4:** Somatic/germline mutations identified in TP53-mutated cases

Cases	TP53 somatic mutation	TP53 germline mutations	Co-existing pathogenic mutations	Co-existing VUS mutations
1	c.614A>G p. Tyr205Cys	–	–	–
2	c.559+1G>A	–	PIK3CA (Glu545Lys)	–
3	c.824G>A p.Cys275Tyr	–	–	KMT2C (Arg2609Gln), RB1 (Asn663Ser), NOTCH1 (Leu818Pro)
4	c.488A>G p.Tyr163Cys	–	–	RAD50 (Arg365Gln), TP53 (Leu188Lysfs^*^59), CCND1 (c.724-2A>C)
5	c.824G>A p.Cys275Tyr	–	AKT1 (Glu17Lys)	–
6	c.818G>T p.Arg273Leu	–	PIK3CA (Glu39Lys)	–
7	c.714_715insT p.Asn239Ter	–	–	ROS1 (Gly2245Ser) ROS1 (Thr145Pro)
8	c.536A>G p.His179Arg	c.847C>T p.Arg283Cys	–	Somatic: ROS1 (Thr2195Ser) TP53 (Arg283Cys) Germline: PMS2 (His189Pro)
9	c.85_86del: p.Asn29GlnfsTer13	–	–	ROS1 (Thr145Pro) BRCA2 (Phe3289Leu)
10	c.586C>T p.Arg196*	–	–	–
11	c.797G>A p.Gly266Glu	–	PIK3CA (Glu545Lys), PTEN (Glu285Glyfs*13)	MSH2 (Glu561Lys), NOTCH1 (Glu606Lys)
12	c.824G>A p.Cys275Tyr	–	–	ROS1 (Thr145Pro), RET (Thr562Ser)
13	c.853G>A p.Glu285Lys	–	–	ROS1 (Thr145Pro), NF1 (Met102Val)
14	c.990del p.Gln331Argfs*14	–	–	MET (Pro1364Ser), NF1 (Ala188Glu)
15	c.742C>T p.Arg248Trp	–	–	ROS1 (Arg167Gln), NF1 (Asp2465Glu), ERBB2 (Arg487Gln), AR (Glu494Ala)
16	c.722C>T p.Ser241Phe	–	PIK3CA (Glu545Lys), NRAS (Gly60Arg)	BRCA2 (Arg1160Gly), BRCA2 (Cys1159Tyr), CDKN2A (Arg10Trp), RET (Glu768Lys), STK11(Val66Met), STK11 (Gly408Ser), NF1 (Ala2485Gly)
17	c.743G>A p.Arg248Gln	–	–	KMT2C (Glu1625Lys)
18	c.638G>T p.Arg213Leu	–	–	KDR (Leu625Phe), CDK4 (Glu265Lys), CDK4 (Met264Ile)
19	c.817C>T pArg273Cys	–	–	ROS1 (Arg1942Trp) KMT2C (Gly4411Arg)
20	c.455C>T p.Pro152Leu	–	–	ROS1 (Thr145Pro), MET (Pro1382Ser), KMT2C (Asp2692Ala) MYC (Asn26Ser)
21	c.743G>A: p.Arg248Gln	–	–	ROS1 (Arg167Gln) KMT2C (Arg1292Gln)
22	c.681_682insT p.Asp228Ter	–	–	MTOR (Arg1896Gln) ROS1 (Gly2245Ser) KMT2C (Ile439Val)
23	c.626_627del: p.Arg209LysfsTer6	–	–	PIK3CA (Gly439Ala) CDK6 (Arg90Thr) JAK2 (Glu1024Lys)

### Survival analysis

**Figure 1:**
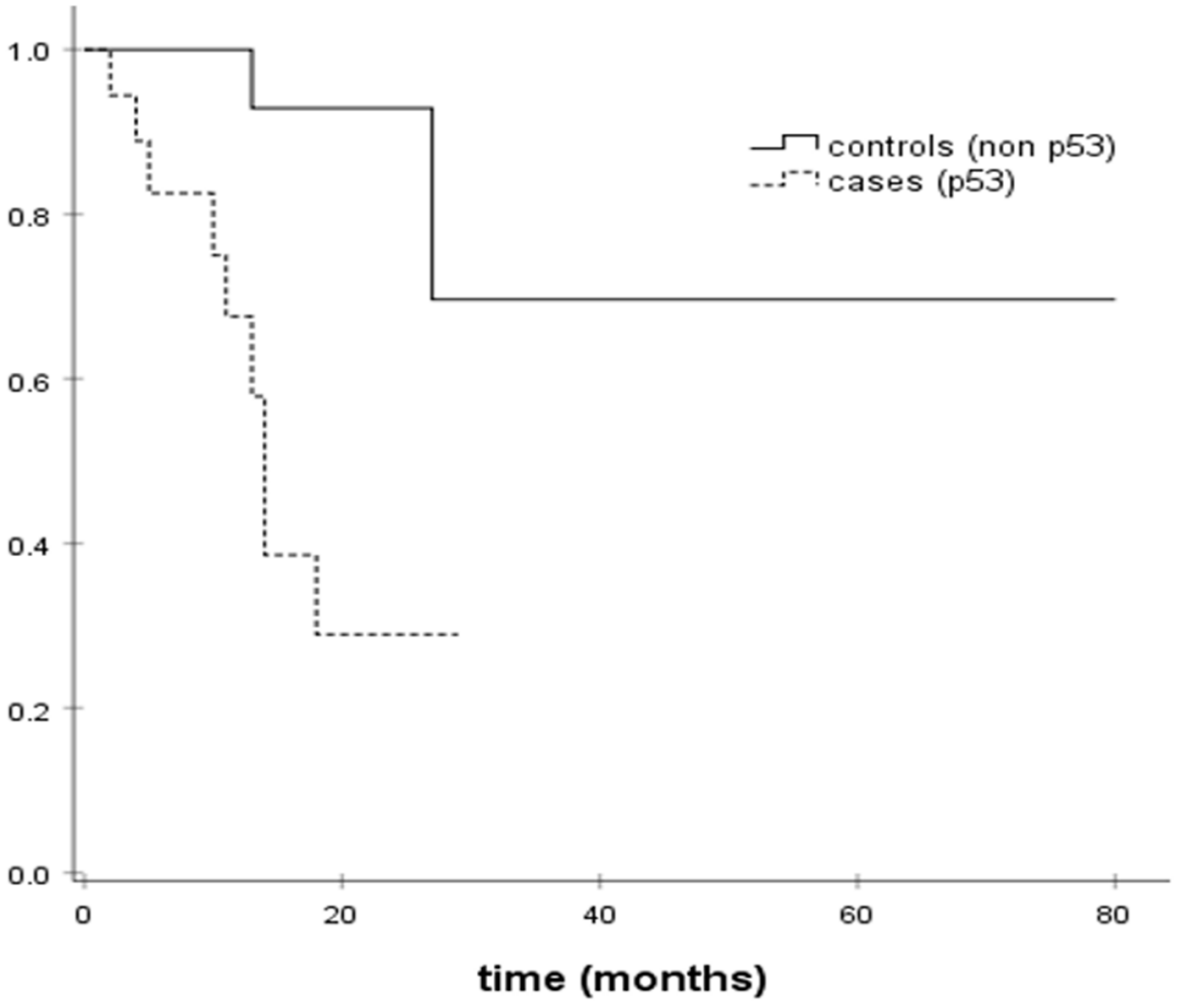
Kaplan–Meier DFS estimates.

Disease-free survival (DFS) was evaluated in cases and controls. DFS was 16.3 months in cases harboring TP53 somatic mutations (95% CI; 11.38–21.25) versus 62.9 months in TP53 wild-type controls (95% CI; 40.8–85). Somatic TP53 mutations were associated with shorter DFS in our study. Figure 1 presents Kaplan Meier DFS curves for cases and controls. TP53 mutation was associated with a 8-fold risk of recurrence in the Cox regression analysis (OR = 8.530, 95% CI: 1.81–40.117; *p* = 0.007).

## DISCUSSION

We here demonstrate that pathogenic somatic TP53 mutations are associated with a decreased disease-free survival in patients with early-stage breast cancer. We retrospectively identified 23 breast cancer patients harboring TP53 somatic mutations identified via next-generation sequencing and 23 TP53 wild-type controls matched on age and clinicopathological characteristics. DFS was significantly reduced in TP53-mutated cases (16 vs. 63 months).

In our study TP53 somatic mutations were associated with high grade (G2/3: 22; 95.7%) and high proliferating (Ki67 > 20%: 87%) breast tumors. This is in accordance with previous results that TP53 mutations are more frequently identified in HER2-positive and triple negative breast tumors (TNBC), while the incidence is low in luminal A tumors. TP53 mutation is indicative of a more aggressive entity and affects tumor response to treatment [15]. P53 regulates epithelial to mesenchymal (EMT) process and stem cell characteristics through upregulation of miR200c [15]. Mutant TP53 tumors suppress transcriptional factors involved in the TGF-*β* signaling pathway (e.g. ZEB2/SIP1, Snail, Twist) and induce epithelial-mesenchymal transition (EMT). Moreover, genes involved in cell migration like the matrix metalloproteinase family member MMP1 were overexpressed in TP53 mutated tumors [15]. Overall, TP53 mutational status was related to reduced cell differentiation and increased metastatic ability.

An interesting finding of our study was the co-existence of TP53 mutations with pathogenic somatic PIK3CA mutations in the 17.4% of the cases. PIK3CA mutations are detected in more than one third of HR-positive breast tumors (34.5%) and less frequently in HER2-overexpressing tumors (22.7%), whereas the incidence drops to 8.3% in triple-negative and basal-like breast cancer [16]. It has been previously shown that co-mutation of TP53 and PIK3CA account for more than 6% of breast cancers and for approximately 30% of TP53-mutated tumors [17]. Co-mutated tumors represent an aggressive entity and were associated with a worse progression-free survival [17]. In our study, co-existence of these mutations was not a rare event. The effect of the simultaneous presence of TP53 and PIK3CA mutations on response to chemotherapy and prognosis needs to be further addressed.

Our case-control study demonstrated that TP53 pathogenic somatic mutations are associated with a reduced disease-free survival in early-stage breast cancer. In agreement with our findings, previous studies report an adverse prognosis in patients harboring TP53 mutations [6]. Some other studies suggest that TP53 mutations have a distinct role in different breast cancer subtypes. TP53 somatic mutations were associated with a worse prognosis in patients with luminal B and HER2-positive breast tumors, but not in patients with luminal A and basal-like tumors [18]. Moreover, TP53 mutations are linked to different response rates to different treatment regimens. One study suggests that TP53 wild-type tumors respond better to hormone therapy, while the opposite effect is identified in patients that receive chemotherapy only [19]. TP53 status may have a distinct clinical role according to the tumor subtype and the type of treatment administered.

One strength of our study was the detection method applied for the identification of *TP53* mutations. DNA sequencing is currently the gold standard for identification of TP53 mutations [20]. Recently, next generation sequencing has emerged as a highly accurate alternative since it offers the ability of detecting mutations outside exons 5–8. Accuracy of immunohistochemistry (IHC) is limited by the presence of null mutations (nonsense mutations, deletions, insertions etc) in TP53 gene that result in a detectable but unstable protein. In these cases that account for up to ~40% of TP53 mutations in breast cancer IHC will fail to detect TP53 mutation. Consequently, IHC detection of TP53 status should be evaluated with caution. Our study overcomes this limitation by applying next generation sequencing in patient samples further increasing the validity of the data presented. Despite the originality, limitations of this case-control study should be acknowledged. Our study is confined to a single institution and thus the sample size is limited. More studies with a larger sample size should be performed to confirm our results. A multicenter study with a similar design could generate more robust scientific data.

We here show that TP53 pathogenic somatic mutations are associated with a shorter DFS in early-stage breast cancer patients. In addition, TP53 mutations often coexist with PIK3CA mutations in breast tumors (17.4%). Future well designed studies should be performed to address the clinical role of the co-existence of these mutations in breast cancer.

## MATERIALS AND METHODS

### Subjects

Incident cases of 82 patients with histologically confirmed Stage I-III breast cancer that underwent NGS in paraffin blocks and blood samples during the period 25/09/2019 to 25/05/2021 were retrospectively collected. Among them 23 cases of somatic TP53 mutations were detected and were matched on age at diagnosis (±5 years), histological subtype (luminal A, luminal B, HER2-enriched, TNBC), histological grade (1 vs. 2/3), menopausal status, ki67 expression and disease stage as classified by TNM classification system with controls; controls included 23 women with TP53 wild-type breast cancer. All women were treated in a single Institute at the Oncology Department of “Alexandra” Hospital, Medical School, University of Athens, Greece. Immunohistochemical (IHC) analysis was performed to quantify expression of human epidermal growth factor receptor 2 (HER2), hormone receptors (HR) and Ki67. Estrogen receptor (ER) and progesterone receptor (PR) were considered positive if tumors had more than 1% nuclear-stained cells. HER2 status was considered positive when graded as 3+, while 0 to 1+ were negative and 2+ was an inconclusive result and *in situ* hybridization (ISH) was performed in those cases to confirm positivity. Hormone receptor positive tumors characterized by ki67 expression of over 20% were considered as luminal B. Information on histological characteristics (tumor subtype, grade, ER/PR expression, HER2 expression, expression levels of ki67), TNM stage (tumor size, lymph node infiltration, metastasis), type of surgery performed, type of chemotherapy administered, disease-free survival and overall survival were collected from patient files and were registered on an electronic database. Both cases and controls were Caucasian and reside in the same geographical region. This case–control study is in accordance with the Helsinki Declaration and has been approved by the Review Board of Alexandra General Hospital of Athens. An informed consent form was obtained from each of the eligible patients.

### DNA extraction

For breast cancer patients, paraffin-embedded breast tissues derived from mastectomy or breast conserving operation before adjuvant treatment and blood samples were analyzed. Paraffin-embedded breast tissues were cut at slices of 10 μm diameter. Tumor DNA was isolated from paraffin-embedded breast tissues using the QIAamp DNA FFPE Tissue or the kit Ion Ampliseq Custom Next Generation Sequencing (NGS) DNA panel (Amplicon Sequencing following the manufacturer’s instructions. Plasma blood samples were collected in Vacutainer tubes. Within 4 hours after collection, plasma was separated from whole blood samples through centrifugation for 10 min at 3000 rpm at room temperature and stored at −80°C until further use. Isolation of plasma DNA was performed using QIAsymphony DSP DNA Mini Kit and the genomic library was constructed using Trusight™ Comprehensive Hereditary Cancer Panel – Nextera™ DNA Flex Pre-enrichment Library Prep according to the manufacturer’s instructions.

### Targeted sequencing

The NGS study on paraffin-embedded breast tissues was performed using Ion Torrent platform (Ion S5Prime) with a median amplicon cover 2000x (whenever DNA extraction was performed with kit Ion Ampliseq). For the data annotation and analysis IonReporter (v5.12) (Thermo Scientific) was used. The sequences were aligned to the human genome reference sequence GRCh37-hg19. An additional manual data curation was performed using data from OncomineReporter (v4.4) and relevant databases (CinVar, dbSNP, Ensemble, COSMIC, CIVIC, PharmGKB, OMIM, My Cancer Genome, Vasome etc.). For the tissues that underwent DNA extraction via the QIAamp DNA FFPE Tissue, libraries were constructed using AmpliSeq for Illumina Comprehensive Panel v3. The NGS study was performed using the Illumina platform (MiSeq, NextSeq500 or NovaSeq) in these cases with a median amplicon cover 500x for the 88.8% of the targeted regions.

### Plasma DNA sequencing

Genomic libraries were constructed using Trusight™ Comprehensive Hereditary Cancer Panel – Nextera™ DNA Flex Pre-enrichment Library Prep according to the manufacturer’s instructions. Plasma sequencing was performed using Illumina platform (NextSeq500/NovaSeq). The validation of results was performed according to criteria of American College of medical Genetics – ACMG [21] and NCCN guidelines.

### Statistical analysis

The statistical analysis was performed with SPSS 24.0 statistical software. Differences between cases and controls were examined by Student’s *t*-test for continuous variables or the chi-square test for categorical variables. Pearson’s correlation and Fisher’s exact test (for categorical variables) were used. The threshold for statistical significance was set at *p* < 0.05. Univariate Cox regression analysis was performed to evaluate the association of TP53 mutation with disease-free survival in breast cancer patients. Kaplan–Meier survival curves were estimated to graphically represent the results.

## Abbreviations

TP53: Tumor protein 53
NGS: next-generation sequencing
PIK3CA: phosphatidylinositol-4,5-bisphosphate 3-kinase, catalytic subunit alpha
DFS: disease-free survival
ER: estrogen receptor
PR: progesterone receptor
HER2: human epidermal growth factor receptor 2
IHC: immunohistochemistry
IDC: invasive ductal carcinoma
VUS: variants of unknown significance
TNBC: triple negative breast cancer
EMT: epithelial to mesenchymal transition
FFPE: Formalin-fixed paraffin-embedded
